# Montane rainforest dynamics under changes in climate and human impact during the past millennia in northern Madagascar

**DOI:** 10.1098/rsos.230930

**Published:** 2024-08-21

**Authors:** Vincent Montade, Laurent Bremond, Helena Teixeira, Thomas Kasper, Gerhard Daut, Sylvie Rouland, Elysée Rasoamanana, Perle Ramavovolona, Charly Favier, Fabien Arnaud, Ute Radespiel, Hermann Behling

**Affiliations:** ^1^ Department of Palynology and Climate Dynamics, Albrecht-von-Haller-Institute for Plant Sciences, University of Goettingen, Göttingen, Germany; ^2^ ISEM, University of Montpellier, CNRS, IRD, EPHE, PSL Research University, Montpellier, France; ^3^ UMR ENTROPIE, University of La Réunion, IRD, CNRS, IFREMER, Université de Nouvelle-Calédonie, Ile de La Réunion, France; ^4^ Institute of Zoology, University of Veterinary Medicine Hannover, Bünteweg 17, 30559 Hannover, Germany; ^5^ Physical Geography, Institute for Geography and Geology, University of Greifswald, Greifswald, Germany; ^6^ Physical Geography, Institute of Geography, Friedrich Schiller University, Jena, Germany; ^7^ Department of Plant Biology and Ecology, Faculty of Sciences, University of Antananarivo, Antananarivo, Madagascar; ^8^ EDYTEM, Université de Savoie, CNRS Pôle Montagne, 73376 Le Bourget du Lac, France

**Keywords:** palaeoecology, late Holocene, drought, fire, demography, bottleneck

## Abstract

Although it is well known that humans substantially altered the Malagasy ecosystems, the timing of the human arrival as well as the extension of their environmental impact is yet not well understood. This research aims to study the influence of early human impact and climate change on rainforests and wildlife in northern Madagascar during the past millennia. Results obtained from the lake sediment in a montane environment showed significant changes in vegetation within the lake catchment associated with a major drought that started approximately 1100 years ago. Human impact, revealed by fires, began at roughly the same time and occurred outside the lake catchment. Although this does not dismiss the impacts that humans had at a regional scale, this result demonstrates that the late Holocene natural drought also significantly impacted the ecosystems independently of anthropogenic activities. At a regional scale, a review of species demographic history revealed a substantial number of population bottlenecks during the last millennia, probably resulting from this combination of human-related impact and natural climate changes. This research highlights the importance of a multi-site and multi-proxy comparison for deciphering the nature and succession of environmental changes.

## 1. Introduction

Tropical rainforests are essential to humankind, for goods, services, carbon sequestration and, therefore, for the balance of global biogeochemical cycles [1]. However, a large part of the rainforest is facing a rapid increase in anthropogenic pressures mainly through deforestation [2]. This is often linked to the unprecedented expansion of human populations and the result of the development of agricultural practices carried out at the expense of rainforest. Madagascar represents a typical example; fast human population growth coincided with accelerated forest loss and fragmentation over past decades [3]. Most remaining rainforests are scattered across eastern Madagascar and in mountain areas containing small rainforest pockets. Since Madagascar is a major hotspot of biodiversity [4], these pockets are critical to protecting its unique biodiversity of forest-dependent taxa, such as the endemic lemuriformes. In addition, these montane rainforests are essential for water resources, as they recycle moisture through the evapotranspiration processes and prevent run-off and soil erosion, thereby making a major contribution to the total water discharges for the surrounding lowlands [5]. While some of the steeper mountain areas in Madagascar remain relatively preserved from human impacts, the anthropogenic alterations are generally more intense on foothills (i.e. at lower altitudes). This difference may be partly explained by the official protection of certain mountain areas but also by climatic conditions (i.e. high rainfall and cool temperatures) and their characteristic steep slopes, which may have slowed down the settling of human populations. To better understand the responses of Malagasy rainforests and biota to future climatic changes, it is critical to study ecological changes based on present-day datasets and on long-time-scale records that extend beyond historical periods [6]. Ecological records from sediment cores covering several millennia help to analyse a broad range of ecological trajectories. Such continuous high-resolution palaeoecological records depend on the availability of humid areas (e.g. lake, bog, marsh), having remained permanently wet through several thousands of years, a condition difficult to meet in regions with highly seasonal rainfall regimes.

**Figure 1.**
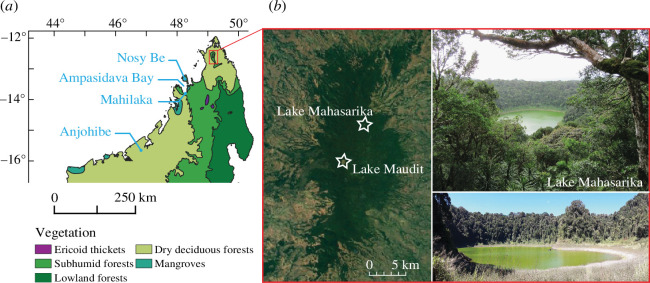
(*a*) Distribution map of natural biomes in northern Madagascar [7]. (*b*) A satellite image of Montagne d’Ambre (source: Google Earth) indicating the location of Lake Mahasarika and Lake Maudit. The two photos of Lake Mahasarika show the tropical forest in the crater and the herbaceous plants (grasses and sedges) restricted to the shores of the lake (source: V. Montade and H. Teixeira).

The Montagne d’Ambre National Park, located in northern Madagascar, represents a highly suitable area to obtain continuous sedimentological records as several crater lakes occur at different elevations within the same mountain area (figure 1). A first study based on sediment archives obtained from Lake Maudit, situated at 1250 m above sea level (a.s.l.) near the mountaintop [8], enabled the reconstruction of environmental changes during the past 25 000 years and provided important insights into the impact of those changes on the local wildlife. This record revealed a strong influence of the African humid period (AHP) between 15 000 and 5500 years ago, as also shown in East Africa [9]. In particular, the increase in temperature and humidity during this period resulted in the replacement of a montane forest by an evergreen humid forest. A subsequent spatial and demographic expansion of the local endemic Montagne d’Ambre mouse lemur was recorded over the same period. Then, the AHP termination led to lower precipitation, triggering the decline of the Montagne d’Ambre mouse lemur. It is plausible that such environmental changes also impacted the distribution and abundance of other forest-dependent taxa, as species directly respond to changes in the available resources [10]. From 1000 years ago, a peat bog has developed at this study site, which currently covers a large part of the lake. At the same time, the occurrence of fires revealed the beginning of human impacts. The latter result is consistent with archaeological data showing an increase in human activities recorded in several regions shortly before the last millennium [11]. However, the increase in anthropogenic activities hardly explains the development of the palustrine and peat bog vegetation, which rather resulted from the filling up of the sedimentary basin and/or a hydrological change. During this critical period of change, anthropogenic activities have been marked by a transition from hunting/foraging to herding/farming in Madagascar, which increased fire frequency and has been interpreted as the main driver of ecosystem alterations and species extinction [12,13]. Nevertheless, the time of permanent human settlement in Madagascar and its role in species declines are still debated [12,14–17]. For disentangling timings and drivers of past environmental changes on vegetation dynamics, a gold standard in palaeoecology is to provide multi-site reconstructions. In this context, our study of a new lake site at a lower elevation in Montagne d’Ambre (Lake Mahasarika, 1073 m a.s.l.) aims to better understand and infer the regional responses, and not purely site-related responses to past environmental changes. More specifically, based on the multi-site comparison, our detailed analysis of late Holocene (the past 4000 years) changes aims to disentangle potential different drivers of environmental and forest dynamics within Montagne d’Ambre. Additionally, the palaeoecological results will be compared with our present knowledge of the corresponding demographic dynamics of wildlife from northern Madagascar.

These new data from Montagne d’Ambre (Lake Mahasarika, lower altitude) compared with available data (Lake Maudit, higher altitude [8]) help to answer the following questions: When did humans start to impact ecosystems in northern Madagascar? To what extent have rainforests and wildlife been impacted locally and regionally? Is there evidence for natural climate changes impacting vegetation changes independently of human impact over the last millennia?

## 2. Material and methods

### 2.1. Palaeoenvironmental study site

Montagne d’Ambre is a volcanic massif reaching up to 1475 m a.s.l., which spreads 35 km north–south and 15 km east–west, located in northern Madagascar at about 10 km south of Antsiranana. In this region, the climate is characterized by heavy rainfalls in austral summer, which are related to the intertropical convergence zone in northern Madagascar when it moves to its southern boundary [18]. The study site, Lake Mahasarika, is one of the six crater lakes distributed at different elevations on Montagne d’Ambre. Lake Mahasarika is located on the northeastern flank of the massif at an altitude of 1073 m a.s.l. (figure 1). This lake is small (250 m wide) and has a small circular catchment area (2.3 km^2^) with steep slopes (>30%) related to the topography of the volcanic crater (figure 1). Local climatic conditions in Montagne d’Ambre differ from the climate at lower elevations. Air cooling with increasing altitude generates frequent fog and important orographic precipitation that reaches above 3000 mm yr^−1^ [19]. This allows the development of a dense and humid rainforest that contrasts with the dry forest and savanna found below 800 m a.s.l. in areas surrounding the mountain [20]. In the lake catchment, a humid rainforest grows all around on the steep slopes while on the shores, where the topography is flattened, seasonal lake level fluctuations allow the growth of herbaceous plants (grasses and sedges) during the dry season (figure 1*b*). The ecotone between the rainforest and the sub-humid/dry forest (approx. 800 m a.s.l.) is located a few kilometres (2–3 km) from Lake Mahasarika (1073 m a.s.l.). However, today the sub-humid/dry forest has almost entirely disappeared [3] due to anthropogenic activities, especially to recurrent fires and cattle grazing.

### 2.2. Sediment coring and age-depth modelling

Fieldwork was carried out in August 2018. Based on bathymetry, the sediment core LMAHA-18 was taken from the central and deepest part of the lake (7 m water depth; 12°32′6.277″ S, 49°10′37.225″ E). Core LMAHA-18 was sampled with a Livingstone piston sediment corer operated from a platform settled on two rubber boats. Using sampling tubes of approximately 5 cm diameter and 120 cm length, a total of six sections of about 100 cm sediment length were sampled with overlaps of 10–50 cm length. Preserved in aluminium tubes for transport to the laboratory, the sediment was extruded from each aluminium tube and split into two sister cores, LMAHA-18a and LMAHA-18b. Each sister core was stored under cool (4°C) and dark conditions at the laboratory facilities (LMAHA-18a at the Friedrich-Schiller-University Jena and LMAHA-18b at the University of Goettingen, Germany). Cores were photographed and magnetic susceptibility was scanned in 3 mm steps with three replicate measurements using an MS2E surface scanning sensor (Bartington Instruments). Using lithological description, patterns of magnetic susceptibility and specific marker layers, the different core sections were aligned to a composite master sequence LMAHA-18 (electronic supplementary material, figure S1). The age-depth model is based on 16 AMS radiocarbon dates of 15 bulk sediment samples and 1 plant macro-remain (electronic supplementary material, figure S1 and table S1), performed at either the Poznań radiocarbon laboratory (Poland) or the LMC14 (Gif-sur-Yvette, France) laboratory. The age of the sediment surface is considered modern and was thus set to the year of coring (AD 2018 = −68 calibrated year BP). The age model was performed as a function of the composite depth with RStudio software using the R-package ‘Bacon’ (v. 4.0.5) [21] and using SHCal20 calibration curve and bomb ^14^C curve [22,23]. The ages are expressed in calibrated years before present (years BP).

### 2.3. Palaeoecological analyses

For pollen extraction, 28 subsamples of 0.5 cm^3^ from core LMAHA-18 were processed following standard chemical techniques with chloridric acid, potassium hydroxide, fluorhydric acid and acetylosis [24]. One tablet of exotic *Lycopodium* spores (20 848 ± 1546, batch number 1031) was added to each sample to estimate pollen concentration. A minimum sum of 300 terrestrial pollen grains was counted for each subsample using a light microscope at 400× magnification, and pollen and fern-spore percentages were calculated on the terrestrial pollen sum. Pollen and spore identification were based on several atlases [25–28], the online African Pollen Database (https://africanpollendatabase.ipsl.fr/#/home), the reference collections from University of Goettingen (http://www.gdvh.uni-goettingen.de/) and the University of Montpellier (https://data.oreme.org/observation/pollen). The main pollen taxa were plotted in the pollen diagram and summarized following the pollen zones identified by cluster analysis (see electronic supplementary material, figure S2 and table S2). Ordinations were carried out to summarize patterns of floristic variation within the tree community changes, independently of the tree/herb balance. Ordinations by detrended correspondence analysis (DCA) were first performed, on terrestrial pollen taxa and on arboreal pollen taxa only, to estimate the underlying linearity of the data. The gradient lengths calculated by the DCA gave values less than two standard deviation units, suggesting underlying linear responses (0.87 and 0.59 s.d. with terrestrial taxa and 0.95 and 0.57 s.d. with arboreal taxa of axis-1 and -2). Accordingly, a linear method, principal component analysis (PCA), was chosen for the ordination [29,30]. By using terrestrial pollen taxa as input variables, non-arboreal pollen taxa (mainly Poaceae) influenced the ordination results (see electronic supplementary material, figure S3). Consequently, the relative abundances of arboreal pollen taxa, defined as the percentage count of each taxon relative to the total number of arboreal pollen grains in a sample, were used as input variables to run the ordinations. In all ordinations, a square root transformation of the pollen percentages was performed to downweight the effect of pollen taxa with high abundances. The ordinations were undertaken using RStudio with the R-package ‘Vegan’ (2.5-7) and ‘Ade4’ (v. 1.7-16) [31,32].

For extraction of charcoal particles, 1 cm^3^ of sediment was taken every centimetre along the LMAHA-18 sediment core. Each sample was soaked in a 3% NaP_2_O_4_ solution plus bleach for several hours to deflocculate the sediment and oxidize the organic matter. The samples were sieved through a 160 μm mesh and the carbon particles were counted using a 40× magnification stereomicroscope.

Bulk organic δ^13^C was measured against certified standards (L-Prolin, EDTA and USG65) and reported in standard δ notation (‰) against Vienna Pee Dee Belemnite (VPDB) and air, respectively. Relative errors based on triplicate measurements are 0.05‰ for δ^13^C. Pollen data are available in the African Pollen Database and palaeoenvironmental data (charcoal particles, magnetic susceptibility and δ^13^C) are available in Pangaea. All the codes for statistical analyses are accessible in Zenodo [33].

### 2.4. Demographic dynamics of wildlife in northern Madagascar

A comprehensive review was conducted on published molecular population demographic studies conducted on Malagasy taxa from northern Madagascar. Studies were collected from the literature using a combination of the following keywords: population genetics, population dynamics, phylogeography, demography, demographic modelling, coalescence, bottleneck, expansion, Quaternary climate change, vegetational shifts and Madagascar. All studies were carefully screened to confirm that the study species were distributed and sampled in northern Madagascar, roughly defined as the area ranging from Nosy Be to the Loky-Manambato region, to achieve the best possible congruence with the palaeoenvironmental records. Studies on species that only occurred outside northern Madagascar were excluded from our review. We reviewed the evidence from studies using different molecular markers (i.e. mitochondrial DNA, microsatellite loci and genome-wide single nucleotide polymorphism) and various demographic approaches. Studies that fulfilled our criteria were evaluated regarding taxonomic representation (i.e. mammal, bird, amphibian, non-avian reptile, plants) and habitat type (i.e. adapted to dry forest, humid forest or both habitats). Finally, the direction of the detected population demographic change (i.e. constant population size, population bottleneck, expansion or size recovery) was assigned to the palaeoecological periods of interest whenever possible. When multiple studies were available for a single taxon, they were all included in the review for comparative reasons.

## 3. Results

The results were divided into three sections to describe the main environmental changes: (i) a PCA carried out on the arboreal pollen taxa to summarize compositional changes in tree community; (ii) the comparison of the palaeoecological and sedimentological results to identify key periods (P1 to P3) of palaeoenvironmental changes; and (iii) a review of the population demographic studies during the past 15 000 years of Malagasy wildlife in northern Madagascar. Detailed results, including the sedimentology, age-depth model, pollen data, charcoal data and summary of the demographic studies currently available for Malagasy wildlife distributed across the north of Madagascar using molecular datasets are available in the electronic supplementary material.

### 3.1. Changes in tree communities

**Figure 2.**
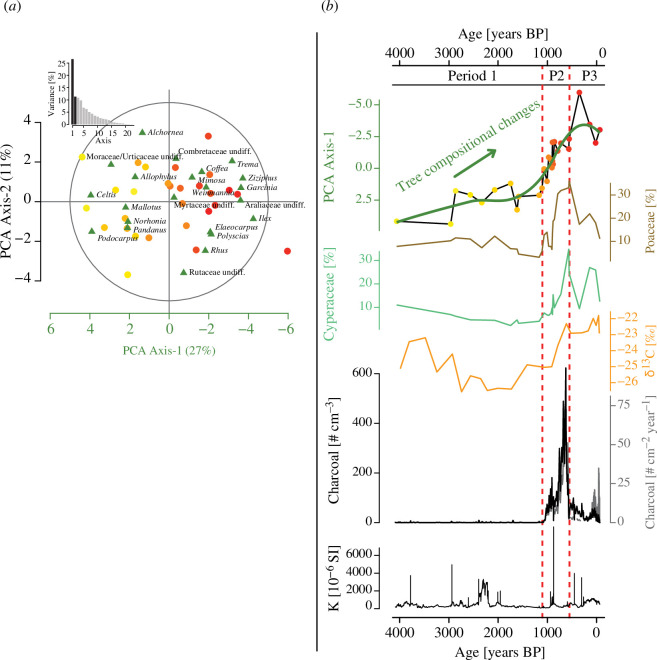
(*a*) Principal component analysis (PCA) of arboreal pollen taxa obtained from the composite sediment core recovered from Lake Mahasarika (LMAHA-18). The *x*- and *y*-scales represent the observation (dots, samples) values on axes 1 and 2, while the variables (green triangles, arboreal pollen taxa) are represented within the correlation circle with values between −1 and 1 following the same axes. The dot colour gradient represents the age of each sample, from yellow for the oldest ones towards red for the most recent ones. The histogram at the top left shows the percentage of total variance explained by each PCA axis. (*b*) Synthetic palaeoenvironmental reconstructions of the composite sediment core LMAHA-18 during the past 4000 calibrated years before present (years BP). From top to bottom are the PCA axis-1 values for pollen samples, percentages of Poaceae and Cyperaceae, δ^13^C measured on bulk organic fraction, concentration (black) and influx (grey) of charcoal particles (>160 μm) and magnetic susceptibility (*k*). The vertical dashed lines (red) demarcate the succession of the three main periods (P1 to P3) of palaeoenvironmental changes defined from the LMAHA-18 lake record.

The PCA resulted in one main axis, the PCA axis-1, that accounts for 27% of the total variance. Along axis-1, *Celtis*, *Podocarpus* and Moraceae/Urticaceae display high positive loadings while *Ilex*, *Ziziphus*, *Garcinia*, Araliaceae and *Trema* display high negative loadings (figure 2*a*). The samples are chronologically distributed along this axis (figure 2*a,b*) illustrating the transition from taxa with positive values (yellow dots) to taxa with negative values (red dots). The sample scores along the PCA axis-1 show a continuous trend towards negative values over the past 4000 years BP with a marked change from 1100 years BP (figure 2*b*). The following three axes account for much lower values of the total variance, around 10% for each of them, then rapidly below 5% for the following ones. While the PCA axis-1 summarizes well the main changes of arboreal pollen taxa over the record, the other axes, as highlighted for the PCA axis-2 (see electronic supplementary material, figure S3), do not represent any consistent pattern. Only the PCA axis-1 was, therefore, considered for the interpretation of tree community changes through time.

### 3.2. Palaeoenvironmental changes

#### 3.2.1. Environmental conditions from 4000 to 1100 years BP (period 1)

During this period, the sediment was dark to light brown, i.e. homogeneous and organically rich. Except for some peaks of magnetic susceptibility, interpreted as event-related deposits (electronic supplementary material, figure S1), the sediment accumulation rate (SAR) was relatively low (0.1 cm yr^−1^) and remained constant showing low erosion. With a relatively low proportion of Poaceae (around 10%; figure 2*b*), the rainforest was well established around the study site, as also indicated by the dominance of arboreal pollen (electronic supplementary material, figure S2 and table S1). However, the slow decline in the PCA axis-1 values (figure 2*b*) reflects some changes in arboreal pollen composition. This change was related to *Noronhia* peaks (24%) at approximately 2550 years BP and a gradual increase in *Mallotus* that reached a maximum (27%) at approximately 1150 years BP (electronic supplementary material, figure S2). Low values of δ^13^C were generally below −24‰ and also support the dominance of C3 forest vegetation around the lake (figure 2*b*). A few charcoal particles were recorded, only occasionally, indicating a very low level of fire activity (figure 2*b*).

#### 3.2.2. Ecological changes and fire increase between 1100 and 550 years BP (period 2)

The sediment had the same texture as in the previous period and SAR remained broadly in the same range with values slightly higher between 0.1 and 0.2 cm yr^−1^ (electronic supplementary material, figure S1). A major vegetation change was recorded and characterized by a two-step increase of Poaceae and Cyperaceae pollen from 1100 and 900 years BP, reaching up to 30% each (figure 2*b*). The abrupt decrease in the PCA axis-1 scores reflects a major change in the tree composition from 1100 years BP onwards (figure 2*a*,*b*). This shift was slightly preceded by a change in forest composition characterized by a synchronous decline of *Mallotus* (<10%) and an increase of *Trema* (>10%) (electronic supplementary material, figure S2). From 1000 to 900 years BP, arboreal taxa related to low disturbances and humid rainforest habitat decreased (e.g. *Celtis*, *Podocarpus*, *Pandanus*) at the expanse of new tree taxa (*Garcinia*, *Ziziphus*, *Ilex*, Araliaceae). New taxa, especially *Impatiens*, started to occur (electronic supplementary material, figure S2). Typical of small shrubs growing in disturbed rainforests and rainforest margins, *Impatiens* also provide evidence for an increase in forest disturbance. As pollination of *Impatiens* is entomophilous (producing less pollen than anemophilous plants), pollen grains of *Impatiens* have a low dispersal probability. These pollen grains were more probably deposited near the parent plants, indicating that disturbances occurred locally in the lake catchment. The δ^13^C values around −22‰ from 900 years BP (figure 2*b*) indicated an increased proportion of local C4 vegetation. This supports that the increase of Poaceae and Cyperaceae recorded by pollen data occurred locally in the lake catchment. Charcoal particles showed a two-step increase in values indicating an increase in fire activity (figure 2*b*). The first increase occurred from approximately 1070 years BP onwards and rapidly reached values above 70 particles cm^−3^ and 10 particles cm^−2^ yr^−1^ with a peak around 900 years BP. The second increase occurred from 800 years BP onwards and reached values above 200 particles cm^−3^ and 30 particles cm^−2^ yr^−1^ with a maximum value of around 600 particles cm^−3^ and 100 particles cm^−2^ yr^−1^ at approximately 630 years BP.

#### 3.2.3. Vegetation and fire dynamics since the last 550 years BP (period 3)

After 550 years BP, sediments turned to light brown and SAR started to increase rapidly from 250 years BP, reaching values above 0.2 cm yr^−1^ at approximately 100 years BP, and then values above 1 cm yr^−1^ during the past century (electronic supplementary material, figure S1). This increase is in line with the expected SAR increase near the core top, as the sediment compaction is reduced. Among the main vegetation changes, values of Poaceae pollen decreased progressively from 30 to 10% and Cyperaceae fluctuated between 25 and 10% (figure 2*b*). Except in one sample, PCA axis-1 scores remained in the same range (figure 2*b*). This shows that the tree composition resulting from the last period was maintained with the dominance of *Mallotus*, *Elaeocarpus*, *Norhonia*, *Trema* and Araliaceae, between 4 and 10%, respectively (electronic supplementary material, figure S2 and table S2). The taxa occurring in the previous period (*Ziziphus*, *Gacinia*, *Ilex*) were still represented with the same range of values between 2 and 4%. The δ^13^C values remained around −22‰, showing a mixture of C3 and C4 plants within the catchment of the lake (figure 2*b*). This supports the relatively high proportion of herbaceous plants after 550 years BP. Although charcoal particles were found in every centimetre of the core showing that fires were occurring frequently (figure 2*b*), the concentration and influx showed a general decrease with values generally below 50 particles cm^−3^ and 10 particles cm^−2^ yr^−1^. During the last century, charcoal influx increased due to SAR increase with values above 10 particles cm^−2^ yr^−1^ and with maximum values around 30 particles cm^−2^ yr^−1^ while charcoal concentration remained in the same range of values.

### 3.3. Demographic dynamics of wildlife in northern Madagascar

**Figure 3.**
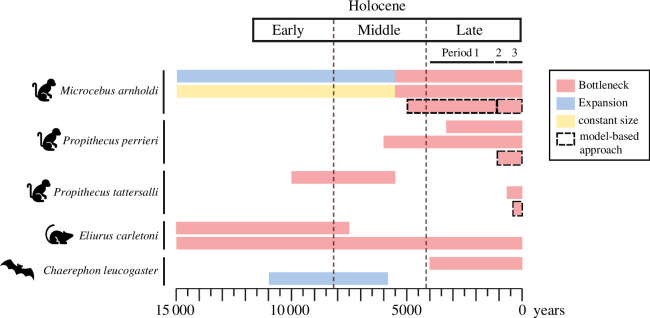
Review results on population demographic dynamics during the past 15 000 years of Malagasy wildlife occurring in northern Madagascar based on molecular datasets (*Microcebus arnholdi* [8], *Propithecus perrieri* [37], *P. tattersalli* [37,38], *Eliurus carletoni* [35,39] and *Chaerephon leucogaster* [40,41]). Only studies with dated demographic events are included in the figure (see electronic supplementary material, table S3, for a review of all the studies currently available). The red, blue and yellow bars represent distinct demographic dynamics (population bottleneck, expansion and constant size, respectively). Dashed bars highlight methods that use model-constrained approaches (i.e. fastsimcoal2 and ABC). The remaining studies rely on model-free methods. The late Holocene was divided into three periods (P1 to P3) following palaeoenvironmental reconstructions obtained from the LMAHA-18 lake record.

A total of 17 demographic studies covering 19 studied species distributed across northern Madagascar fulfilled our criteria and were considered here (electronic supplementary material, table S3). The studies were biased towards mammals (47.4%) and non-avian reptiles (36.8%), with the remaining taxa (amphibians, birds and plants) being represented by a single species each (5.3%). For non-avian reptile species, the timing of the demographic event is either unknown (*Phelsuma dorsivittata*) or dated back to the late Pleistocene (>12 000 years BP), precluding conclusions about recent demographic dynamics. Studies on mammals, however, provide important insights into recent demographic dynamics in Malagasy wildlife. Except for three species for which demographic events were not dated (*Microcebus tavaratra* [34], *Eliurus tanala* [35] and *Microgale brevicaudata* [36]), all other studied terrestrial mammals (i.e. lemurs and rodents) suffered a population decline predating or during the late Holocene (i.e. before or during period 1; figure 3 and electronic supplementary material, table S3). A reduction in population size was also reported for *Chaerephon leucogaster* [40], a bat species, during the late Holocene (period 1; but see [42]). Notably, demographic modelling with the composite-likelihood method implemented in fastsimcoal2 [43] for *Microcebus arnholdi* [8] revealed two consecutive population bottlenecks, one predating period 1 (approx. 5000 years BP) and one during period 2 (approx. 1000 years BP). Demographic modelling with the approximate Bayesian computation (ABC) [44] for two additional lemur species (i.e. *Propithecus perrieri* and *P. tattersalli* [37]) also detected a population decline within the last millennium (period 2; figure 3).

Species demographic response to past environmental versus human pressures may differ among ecological specialist versus generalist taxa. The reviewed studies revealed that all mammals adapted to dry habitats exhibited signatures of a population decline (*Propithecus perrieri*, *Eliurus carletoni*, *Chaerephon leucogaster*; electronic supplementary material, table S3). Except for *Furcifer pardalis* (that underwent a population expansion in the late Quaternary [45]), all taxa adapted to humid habitats also underwent a population bottleneck or kept a constant population size (*Microcebus arnholdi*, *Eliurus tanala*, *Mantella crocea*, *Phelsuma dorsivittata*). In contrast, no clear demographic trend was evident in species adapted to both dry and humid habitats (*Microcebus tavaratra*, *Propithecus tattersalli*, *Microgale brevicaudata*, *Myotis goudoti*, *Dicrurus forficatus*, *Laliostoma labrosum*, *Calumma boettgeri*, *Geckolepis maculata*, *Mimophis occultus*, *Zonosaurus madagascariensis*), suggesting that ecological specialized taxa are more prone to environmental changes than generalist taxa.

Except for two studies [8,37], all demographic studies assumed that the population structure was negligible, and applied either a single demographic approach (statistical tests or a model-free approach) or a Bayesian skyline plot (BSP) coupled with mismatch distribution/neutrality tests. For *Microcebus tavaratra* [34] and *Eliurus tanala* [35], the neutrality tests detected a population expansion, while BSP suggested a constant population size. The neutrality tests were also inconclusive for *Eliurus carletoni* [35]. In contrast, the demographic methods (i.e. model-free and model-constrained approaches) implemented by Salmona *et al*. [37] and Teixeira *et al*. [8] revealed a synchronous population bottleneck for *Microcebus arnholdi*, *Propithecus perrieri* and *P. tattersalli* during the late Holocene (periods 2 and 3).

## 4. Discussion

### 4.1. The period before permanent human settlement in northern Madagascar

**Figure 4.**
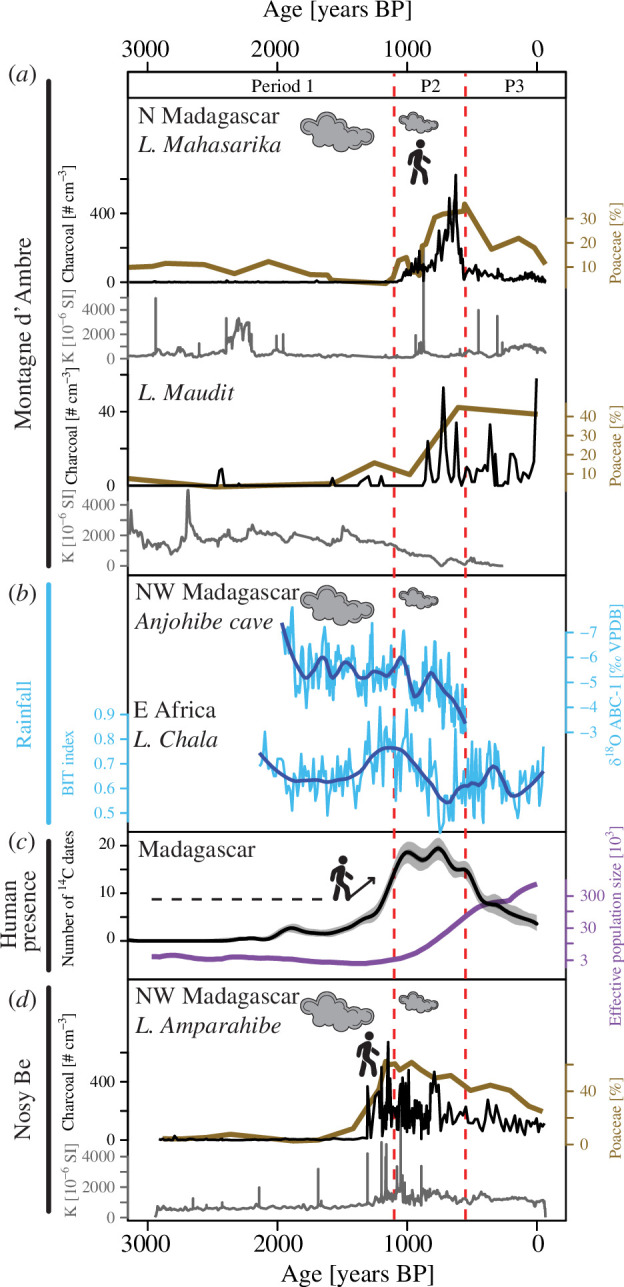
Synthesis of environmental changes on northern (N) and northwest (NW) Madagascar and comparison with East Africa during the past 3000 calibrated years BP (years BP). (*a*) Poaceae percentages, charcoal concentrations and magnetic susceptibility (*k*) from Lake Maharika (LMAHA-18, this study) and Lake Maudit sediment core [8]. (*b*) Decadal-resolution time series of δ^18^O speleothem profile from the Anjohibe cave [18] and BIT index variability from Lake Challa [51] showing precipitation changes in northwest Madagascar and East (E) Africa (the dark blue line represents a smooth spline). (*c*) The black curve represents the number of radiocarbon dates performed on archaeological samples obtained from an archaeological data synthesis [11]. The purple curve represents the estimated changes in the effective human population size [52]. (*d*) Same as (*a*) from Lake Amparahibe [53]. Clouds represent precipitation and the size is proportional to the level of precipitation. The vertical dashed lines (red) demarcate the succession of the three main periods (P1 to P3) of palaeoenvironmental changes defined from the LMAHA-18 lake record.

The exact schedule for the arrival and settlement of humans in Madagascar is not yet clear (e.g. [11,14,16]). Recent evidence for the early to mid-Holocene (>5000 years BP) arrival of humans on the island is solely based on indirect indicators (e.g. cut-marks performed on bones of hippopotamus and elephant birds [16]), and no archaeological artefacts have so far been found associated with these findings. This suggests that even if humans were already inhabiting Madagascar during these early times, they seem to not have impacted the ecosystems sufficiently enough to leave traces in the studied palaeoecological records. The clear first signals of permanent human settlements on Madagascar are generally recorded for the period between 2000 and 1000 years BP (e.g. [11,46,47]). The earliest proof of human civilization with town establishment in Madagascar has been dated at approximately 1000 years BP and it stems from the harbour of Mahilaka in the northwest [48]. Located at the end of a bay, Mahilaka was inhabited by a Muslim population and was connected to the Comoro Islands and probably East Africa by maritime trade routes [48–50]. This population seems to be related to the westward Austronesian expansion that had developed maritime trade and agricultural practices based on rice cultivation in the region from 1250 years BP onwards [50]. A sharp increase in fires from 1300 years BP on Nosy Be Island, 40 km in a straight line from Mahilaka, supports the establishment of agriculture and a significant development of human populations in this region (figure 4) [53]. New results from Lake Mahasarika combined with previously published data from Lake Maudit show very low occurrences of charcoal before 1100 years BP (figures 2 and 4). The charcoal particles counted in these two sites correspond to sedimentary macro-charcoal particles with a size above 160 µm. Not being transported over long distances (maximum few kilometres [54,55]), results suggest that permanent human settlements combined with anthropogenic burning did not exist near Montagne d’Ambre before 1100 years BP. If humans were present at that time, they had only a small impact on ecosystems, at least not strong enough to be visible with the palaeoecological data. In this context, vegetation dynamics before 1100 years BP was most probably caused mainly by natural climate change. Mid- to late Holocene climate in this region is characterized by a multi-millennial drying trend combined with frequent occurrences of megadroughts [18,56]. Sedimentological changes evidenced from the Lake Maudit record, located at a higher altitude in Montagne d’Ambre (1250 m a.s.l.), are consistent with a regional precipitation decrease that intensified at the end of the AHP from 5500 years BP onwards [8]. Starting from 4000 years BP, new pollen data from Lake Mahasarika reveal that dominant tree taxa were progressively changing (figure 2) with *Mallotus* that increased until 1100 years BP. *Mallotus* might be an indicator of forest disturbance [57]. Its increase may show that the rainforest adapted to more drought-related disturbances due to reduced rainfall. The available demographic studies for northern Madagascar showed that all mammals, birds, amphibians and non-avian reptiles ranging from Nosy Be to the Loky-Manambato region exhibit a signature of a population size change (expansion or decline) pre-dating the late Holocene, supporting major environmental changes in the region long before humans impacted these ecosystems.

### 4.2. Human impact and environmental changes in northern Madagascar

From 1100 years BP, the permanent occurrence and gradual increase of charcoal particles combined with the development of grasses and sedges, evidenced by the Mahasarika Lake record, show a major increase in fires (figures 2*b* and 4). Such a change is contemporaneous with the development of human activities in northwest Madagascar, where the first anthropogenic burning started 200 years earlier on Nosy Be Island [53]. In this context, the increase in fires evidenced by the Mahasarika Lake record is also probably related to the beginning of anthropogenic burnings in northern Madagascar. Fires would have increased gradually, inducing changes in forest composition and the development of herbs when the highest fire activity occurred. The question to address is the location of these fires, whether inside or outside the lake catchment area. The increase of δ^13^C values, showing an increased proportion of C4 plants (grasses and sedges) [58], consistently with the rise in sedge and grass pollen, attests to the local development of herbaceous plants in the catchment of the lake, as organic matter in the sediment comes from the lake catchment area. Were this rise in herbaceous vegetation the result of significant forest opening and conversion to savanna by fires inside the catchment area, and considering the small size (approx. 2.3 km^2^) and the steep slopes (>30%) of the catchment area, this would also have resulted in high soil discharges into the lake. Such a process is very well documented from Lake Amparihibe on Nosy Be Island [53]: first peaks and increases in charcoal particles occurred simultaneously with peaks and increases in magnetic susceptibility because soil previously protected by forest vegetation suddenly became prone to erosion (figure 4*b*). However, at Lake Mahasarika, the increase in charcoal particles was not associated with any change in magnetic susceptibility, and even when charcoal particles reached maximum values, magnetic susceptibility remained low. This points to fires occurring near the catchment area but not inside. Such a pattern is observed today; fires do not occur in the catchment, dominated by rainforest except on the shore where grasses and sedges are growing, but outside the catchment a few kilometres away. Charcoal particles found in the most recent sediments (i.e. in the first top centimetres of the core) are therefore transported by winds in the catchment area. Without fires occurring in the catchment, the local past vegetation dynamics might be, therefore, better explained by a lake level decrease allowing the development of grasses and sedges (reaching maxima between 900 and 550 years BP) on the shores of the lake. The complementary results obtained from Lake Maudit (situated 200 m above Lake Mahasarika; figure 1) showed the same overall pattern [8]: an increase in grasses, sedges and fires without any increase in erosion, also suggesting a lake level decrease from approximately 1000 years BP (figure 4). The contemporaneity of these changes, highlighted in these two lakes, was probably linked to a regional precipitation decrease. The precipitation decline would also have led to changes in forest composition (figure 2*b*). As shown by the Mahasarika Lake record, a maximum of *Mallotus* followed by an increase of *Trema*, both typical of trees growing in disturbed rainforests [20,57], slightly preceded the fire increase and might be related to this precipitation change. Although it remains to be confirmed using independent palaeoclimatic reconstructions, this result is consistent with a major precipitation decline or a megadrought evidenced, around 1000 years BP, by palaeoclimate reconstructions in northwest Madagascar [18,56,59,60], the Island of Rodrigues [18] and East Africa [51]. Changes in subsistence strategies with populations shifting from hunter–gatherers to herding–farming have been proposed as a potential explanation of the major ecological changes around 1000 years BP in Madagascar [12,13,61,62]. However, considering the strong hydroclimatic change during this period and without additional palaeorecords combined with archaeological data to obtain a robust synthesis in different regions, the causal links between the observed changes, in particular, during the critical period of transition, cannot be fully clarified at the island scale. In particular, the debate remains open as to whether anthropogenic burning increased in response to precipitation decrease leading the human population to use and develop new subsistence strategies and/or colonize new areas. On the other hand, a decrease in precipitation may have promoted anthropogenic fire expansion and the occurrence of disasters such as mega-fires.

A combination of natural and anthropogenic factors may, subsequently, have led to the dramatic ecological changes observed in northern Madagascar, such as additional animal population declines evidenced by demographic modelling for *Microcebus arnholdi* (Montagne d’Ambre), *Propithecus perrieri* (Analamerana and Andrafiamena) and *P. tattersalli* (Loky-Manambato; reviewed here) or megafauna extirpation, as revealed by archaeological data [63,64]. Although a wide range of approaches are currently available and were used to reconstruct species demographic history from molecular data (e.g. neutrality tests, model-free and model-based approaches), the inference of recent population bottlenecks during the last millennia was only successful with model-based approaches, such as fastsimcoal2 and ABC. Moreover, while some studies, implementing mismatch distribution/neutrality tests and Bayesian skyline plots, detected different demographic trends, the studies implementing model-free and model-based approaches [8,37] revealed congruent dynamics. Altogether, our demographic review confirms that model-based approaches are a very valuable tool to detect recent demographic changes that are not detected by other demographic approaches, and highlights the importance of using more than one method for demographic inferences.

### 4.3. Period of the last 550 years BP

Over the past few centuries, data from Lake Maudit have not shown particular changes and vegetation dynamics but instead mainly reflected the development of a large local peat bog [8]. In contrast, from 550 years BP onwards at Lake Mahasarika and, starting from approximately 200 years earlier at Nosy Be, the proportion of grasses decreased in congruence with a decrease in fires. This pattern, particularly the decrease in fires, might be explained in several ways: first, starting approximately 600 years BP, archaeological data suggested a human population decline in the northwest at Mahilaka and the surrounding villages of Ampasindava Bay [48,65]. Although this remains to be confirmed at the local and regional scale around Montagne d’Ambre with archaeological data, a decline in human occupation in northern Madagascar could have resulted in a reduction of anthropogenic burning. Second, several precipitation records have revealed an increase in humidity from about 500 years BP onwards [59]. This may have reduced the risk of catastrophic fire events, such as mega-fires, and may also explain a general reduction in fires. Third, in case northern Madagascar was entirely forested, the abrupt increase of fires at a regional scale at the beginning of the last millennium might have produced large amounts of charcoal particles. Subsequently, and as a consequence of more frequent fires, fuel availability for large fires should have decreased, which could also explain the observed decrease in carbon particles emitted. These different hypotheses, or a combination of several of them, seem likely but require additional archaeological and palaeoecological data to be tested. Finally, during the last century, a renewed increase in fires is recorded in the Mahasarika sediment core (figure 2*b*). Additional study sites are necessary to confirm this trend that is evidenced by charcoal influx. A fire increase during the last century might reflect the effect of the European colonization in the Montagne d’Ambre region, where Jofreville was established with the development of agriculture from the 1900s [66].

## 5. Summary

This multi-proxy and multi-site comparison of lacustrine cores, located in the north of Madagascar in the mountain range of the Montagne d’Ambre, supports a precipitation decrease, starting approximately 1100 years ago, causing a significant drop in the water level at the two lacustrine study sites (Lakes Mahasarika and Maudit). Under low lake levels, herbaceous plants (mainly grasses and sedges) started to develop on the shores of the two lakes and the decrease in precipitation partly changed the forest composition. Recorded roughly at the same time, anthropogenic burnings spread throughout the study region, but were probably limited at lower altitudes, as it is observed today. Therefore, very likely fires were not the driving force behind these observed changes in the lake catchment areas. Although this does not dismiss the importance humans had subsequently in terms of impact on ecosystems, this work demonstrates that the natural drought that intensified regionally about 1000 years ago significantly impacted the ecosystems independently of anthropogenic activities. The increasing number of inferred population bottlenecks of wildlife at a regional scale during the last millennium, as evidenced by the review of demographic studies, probably resulted from the combination of both human-related impact and environmental changes (i.e. precipitation decline). The remaining important questions, therefore, are not just how precisely humans have influenced their environments, but also whether the natural precipitation decrease caused humans to change their subsistence strategies and/or to move to new areas. While forest loss has increased in recent decades due to an increase in human activity across the island, these palaeoecological data show that these pockets of mountain rainforest have been able to maintain themselves despite important disturbances over the past millennia. Continuing to protect such areas, therefore, seems very relevant and essential in the context of global warming. Finally, this work shows that multi-proxy approaches and multi-site comparisons provide essential evidence for distinguishing the different factors driving environmental changes to better understand ecosystem functioning over the long term.

## Data Availability

All datasets produced in this study have been deposited in public repositories (Pangaea and African Pollen Database). In addition, the script of statistical analyses is available via Zenodo [33]. Supplementary material is available online [67].
